# Supplementary material for: Chromothripsis during telomere crisis is independent of NHEJ, and consistent with a replicative origin
Source: Genome Res. 2019 May;29(5):737–49. doi: 10.1101/gr.240705.118 (PMC6499312; doi:10.1101/gr.240705.118)
Supplement: Supplemental Material [file supp_gr.240705.118_Supplemental_file_1.zip › contigs/annotated_contigs/DB75/contig.2.DB75_length_293_mean_cov_8.69283276451.docx]

**DB75_length_293_mean_cov_8.69283276451**

```
CAGCTTTACCTCTCATAAAACAATGTTATGAAAATTTCCAAATATTTACAATGCCAGACAAAGCCCTACATGATCTGGCTTCTACCTAC
 >chr2:124191631-124191778 - E=3e-68 p=0e+00
CACTCCATCTTCCTCTTGCACTAAAACACATCTCTCTCTCT|CACACACACACACATAC|ATATACACACACACACTTAACCAAGGAGG
                                          >chr4:153746131-153746297 + E=2e-78
CAAAAAATCTTCACAAGGAGAACTACAAAACACTGAAAAAAATAATAGATGACACAAACGAATGAAAATACATTCCATAAGAATCTGTA

TTCTGAAAACGACCATACTGCCCAAAGA
```
